# Accounting for the Opportunity Cost of Children's Time in Economic Evaluation: Challenges and Frequently Asked Questions

**DOI:** 10.1002/hec.70089

**Published:** 2026-03-10

**Authors:** Lazaros Andronis, Cameron Morgan, Cam Donaldson, Emily Lancsar, Stavros Petrou

**Affiliations:** ^1^ Centre for Health Economics at Warwick Warwick Medical School University of Warwick Coventry UK; ^2^ Yunus Centre for Social Business and Health Glasgow Caledonian University Glasgow UK; ^3^ National Centre for Epidemiology & Public Health Australian National University Canberra Australia; ^4^ Nuffield Department of Primary Care Health Sciences University of Oxford Oxford UK

**Keywords:** children and young people, monetary valuation, time inputs

## Abstract

Economic evaluations carried out from a societal perspective ought to account for the opportunity cost of a range of resources, including those committed by care recipients. People's time is such a resource: it is limited, valuable and it has an opportunity cost that should be reflected in cost calculations. Yet, when it comes to children and young people (CYP), there are few suggestions on how to value their time and include it in economic evaluations. Despite repeated calls for research, this remains a persistent gap in our methodology “playbook”. In this paper, we look at the topic by bringing together seminal literature and recent research findings. We discuss key uncertainties that need to be resolved for the topic to move forwards, outline challenges and “frequently asked questions”, offer our views on possible answers and solutions, and sketch out a roadmap for future research.

## 1 Introduction

Economic evaluations are increasingly called on to inform decisions about the adoption of health care programmes offered to different populations, including children and young people (CYP) (Currie et al. 2009). This has resulted in efforts to ensure that available methods for economic evaluations of interventions for CYP are fit‐for‐purpose, and has led to numerous advances that have found their way into our methodology playbook (Ungar and Gerber 2010). Yet there still exist gaps and uncertainties (Andronis et al. 2019; Neumann et al. 2016).

In analyses that adopt a societal perspective, it is important that all salient inputs, including those contributed to or borne by individuals, are measured and accounted for. Patients' time—be it adults' or CYP's—is such an input: time is limited, valuable, and its use in a particular way entails an opportunity cost (Dranove 1996; Posnett and Jan 1996). Given this, seminal textbooks recommend that patient time contributions while seeking and receiving care should be reflected in cost calculations (Drummond et al. 2005; Gold et al. 1996; Neumann et al. 2016), particularly when comparing care programmes that differ in the time demands placed on patients (Russell 2009). This is especially relevant in cases where new interventions, technologies, or care pathways lead to quicker diagnosis (e.g., through point of care diagnostic testing), fewer or shorter appointments (e.g., through streamlined pathways that allow diagnosis, consultations and treatment planning to take place in a single visit) or remove the need for frequent hospital visits (e.g., through combined appointments for multiple services, remote management or virtual consultations and home‐based care). Over the last 30 years, discussions (and debates) on how best to measure, value and account for patients' time costs have led to research that has produced valuable insights (W. Brouwer et al. 2022; W. B. Brouwer et al. 1998; Krol and Brouwer 2015; Rice 1967; Sendi and Brouwer 2004; Tranmer et al. 2005; Zhang et al. 2011) and useful empirical findings (Borisova and Goodman 2003; Hoefman et al. 2019; van den Berg and Ferrer‐i‐Carbonell 2007; van den Berg et al. 2017; Verbooy et al. 2018). However, these focus—almost entirely—on working‐age adults. One will find few insights into, and even fewer recommendations on, how best to value and take into account CYP's time in economic evaluations.

In this paper, we set out to discuss challenges around, and possible solutions to, conceptualizing, valuing and including CYP's time inputs into economic evaluations. To do so, we draw on relevant literature in economics and health economics, on questions and points that have emerged from discussions at conferences and seminar presentations, as well as findings of recent research on the topic (Andronis et al. 2023). The rest of the paper is structured as follows. The next section provides some background: we summarize how individuals’—largely, adults’—time has been perceived and treated in economic evaluations, and we look at whether and to what extent existing thinking and tools can be used to account for CYP's time in such studies. We then move on to discuss “frequently asked questions” on this topic, paying particular attention to “thorny” normative questions and issues that have attracted limited attention. We pinpoint challenges in answering these questions, offer possible solutions, and close by putting forwards an agenda for future research.

## 2 Time in Economic Evaluation

Time as a resource has attracted considerable interest in the economic literature (Gronau 1977). Being limited and valuable, people's time is an economic good that impacts on numerous decisions made by households and individuals (Becker 1965; DeSerpa 1971). Expectedly, this recognition has led to research on various aspects of the topic, including ways to record how people allocate their time across different activities (Gershuny and Sullivan 2019), preferences and determinants that influence such decisions (DeSerpa 1971), as well as the (monetary) value that can be attached to people's time (Grossman 1972). Research on the latter has been pursued in different fields of enquiry, including health, labor and transportation economics, often to underpin economic evaluations of policies or programmes that are likely to lead to changes in an individual's available time (Koopmanschap and van Ineveld 1992; Mackie et al. 2001; Shaw 1992).

Carrying out an economic evaluation involves identifying, measuring and valuing the required inputs into, and the resulting outcomes of, alternative activities competing for funding. Inputs represent the opportunity cost of used or forgone resources and, in health care, outputs typically capture changes in one's health or wellbeing (Drummond et al. 2015; Gold et al. 1996). The exact categories of inputs to be considered depend on the perspective that the evaluation adopts. When this is a wide, societal perspective, it is important that changes in individuals' time are identified, valued and taken into account (Dranove 1996; Russell 2009).

Within the context of an economic evaluation, such changes may largely emanate from care recipients' input into an intervention (e.g., spending time traveling to and attending appointments or engaging in health‐promoting activities) (Gold et al. 1996; Posnett and Jan 1996). This has often been referred to as “patient time” or “time in treatment” and has been seen as a personal cost incurred by patients due to their input into “operationalizing” an intervention (W. B. Brouwer et al. 1998; Gold et al. 1996). As Posnett and Jan (Posnett and Jan 1996) explain *“patients” time is an essential input into all forms of health care provision, and in this sense any evaluation of alternatives characterized by differences in time intensity of treatment will need to take into account of the opportunity cost of patients“time”*.

Changes in time can also occur as a result of the impact of an intervention on an individual's health status. Such changes are typically due to reduced morbidity (e.g., avoiding illness or quicker return to health) or mortality (e.g., extended survival). Insofar as an intervention can affect one's health and, as a result, alter the amount of time one can devote to different paid or unpaid activities, this has been seen as an indirect cost of disease (Koopmanschap and van Ineveld 1992) and has been largely viewed as loss of productivity (W. B. Brouwer et al. 1997; Gold et al. 1996). Productivity costs—defined as “*costs associated with production loss and replacement costs due to illness, disability and death of productive persons, both paid and unpaid.”* (W. B. Brouwer et al. 1998)— have been the focus of much of the literature (and debates) on the broader topic.

Understandably, there is a degree of ambiguity around different—and often overlapping—concepts of time in economic evaluation. To many, this is a nebulous area where “patient time” costs and productivity costs are used synonymously. However, making a distinction between time displaced due to engaging with a treatment and time forgone due to ill health is conceptually useful: the former reflects an input into an (assessed) intervention while the latter is an outcome of the intervention. This distinction is also practical, not least because combined with other factors (e.g., the perspective of the evaluation) it determines how time costs should be conceptualized in an economic evaluation.

## 3 From Hours to Monetary Values

Economic value is a multi‐faceted concept, with different definitions invoked for different purposes. These include the labor value of a good (determined by the amount of labor required to produce the good), the exchange value of a good (defined by the price that a good commands in markets) and the subjective value of a good. Through consumer choice theory, the latter links a good's value to an individual's choices that are assumed to reflect preferences formulated with a view to maximizing utility. On this basis, and since the seminal work of Becker (Becker 1965) and DeSerpa (DeSerpa 1971), consumer theory and utility maximization have offered a foundation for connecting preferences for activities individuals choose to spend time on to utility and, subsequently, to an individual's (subjective) value of time (Evans 1972; González 1997). Conceptually, identifying the value derived from spending time on an activity is a first, necessary step for determining the opportunity cost of forgoing this activity. If, for example, receiving care displaces additional leisure time (or paid work), the opportunity cost of this time ought to reflect the loss of utility due to not being able to spend time doing something else (e.g., watching a movie or generating income).

Traditionally, displaced time has been looked at from the viewpoint of adults' absence from paid or unpaid work, largely as a result of morbidity or mortality. This viewpoint was developed and adopted in early cost‐of‐illness studies (Rice), which sought to estimate the economic impact of a condition on society. Proposed approaches to valuing such time present some variation, but they share a common characteristic: they advocate the use of wages observed in the labor market as an exchange rate that allows converting units of time into costs. Different approaches make different assumptions on whether and to what extent loss of productivity may be compensated for by other (employed or unemployed) persons (Koopmanschap and Rutten 1996; Koopmanschap and van Ineveld 1992; Weisbrod 1961; Dranove 1996) While using wage rates to represent the value (or opportunity cost) of one's time has been seen as simplistic, the approach offers a pragmatic and convenient solution. Wage rates and the value of time, however, are hardly equivalent (Borisova and Goodman 2003; Cauley 1987). In practical applications concerned with significant lengths of time (e.g., years or decades), little consideration is given to time other than “market productive” time (e.g., leisure time), conceivably for pragmatic reasons.

Changes in time spent receiving treatment have received less attention, perhaps owing to the fact that such time tends to be shorter in length and, therefore, resulting changes are perceived to be less consequential (Andronis et al. 2023). Similarly, early literature suggests approaches that use prevailing wage rates in the labor market to value changes in time in treatment (Neumann et al. 2016; Posnett and Jan 1996), though the same criticisms apply, even more so given the shorter length of displaced time. Suggestions that time should be valued differently according to how this time would have been spent otherwise (e.g., in leisure or unpaid work) have introduced useful granularity (W. B. Brouwer et al. 1998). Clearly, approaches to valuing loss of time are geared toward working‐age adults and, for various reasons, they are unsuitable for valuing CYP's time. Fundamentally, most CYP have no observable wages. In most countries, international conventions and legislation prohibit or limit CYP (particularly younger children) from engaging in paid employment and, instead, laws mandate that they attend full‐time education until a particular age is reached.

The fact that our methods for valuing CYP's time currently fall short of addressing this issue (and other related uncertainties discussed below) has led to repeated calls for research (Andronis et al. 2019; Gold et al. 1996; Neumann et al. 2016; Tranmer et al. 2005). Nonetheless, resolving these uncertainties requires overcoming a key stumbling block: what monetary value should the analyst assign to a length of CYP's time? Findings from an international survey of health economists showed that the lack of estimates of the opportunity cost of CYP's time is the most cited reason for not accounting for CYP's time in economic evaluations (Andronis et al. 2023). To a great degree, such views are not surprising; they largely echo the sentiment expressed by the 1st Washington Panel (Gold et al. 1996): *“For adults unable to work and for children in general, there are no wage data. We encourage research into how to better value the time for these two groups.”* The fact that the same call for research was repeated, word‐by‐word, by the 2nd Washington Panel (Neumann et al. 2016) 20 years after the first publication underscores the limited progress on the topic and highlights a pressing need for research (Andronis et al. 2023).

## 4 Issues, Challenges and Frequently‐Asked Questions

Addressing these gaps will require tackling various challenges and answering a number of questions. Below, we highlight key uncertainties and issues that have emerged from our own research (Andronis et al. 2023), as well as pertinent questions that frequently arise in discussions on the topic.

### 4.1 At What Age is a Child's Time Valuable?

A frequently encountered question is whether the value of CYP's time varies with age, and if so, at what age a child may be old enough for the opportunity cost of their time to be considered a meaningful quantity in an economic evaluation. To put this in context, imagine a new intervention for CYP that requires less frequent follow‐up appointments and saves 24 h of a care recipient's time over 3 weeks. Should the decision to include the opportunity cost of this time (over and above any additional cost savings accruing to health care providers and/or parents) depend on the recipient's age? If so, what should be the cut‐off age below which displaced CYP's time should be disregarded? Most respondents in a survey of health economists suggested an age between 4 and 6 years as a cut‐off range, most often justifying this on the basis of CYP's time having a discernible opportunity cost once they start formal education. Others suggested that this cut‐off point should be the stage at which CYP are independent enough to “have a say” in how they spend their own time, or are seen as “economic agents”. On this, some empirical evidence shows that children are increasingly involved in making choices (either in communication with parents or by making their own decisions) after they reach primary school (Lundberg et al. 2009). While insights from various studies within and beyond our field are useful, this is largely a normative question that, ultimately, requires a value judgment.

### 4.2 How Should the Value of Changes in Time Be Accounted for in Economic Evaluations?

A further question we have encountered frequently relates to how changes in CYP's time may be best reflected in an economic evaluation. Should this be translated into monetary terms and included on the “cost” side of an evaluation, or should it be presented as an outcome, reflecting the intervention's output? To answer this question, it is useful to consider how changes in time are brought about: due to spending more (or less) time receiving treatment or due to having more (or less) healthy time as a result of the treatment per se. As long as this is made clear, the basic principles of economic evaluation prescribe how these should be treated.

To put this in context, imagine that, in the case of the former, a new intervention reduces the number of appointments needed to treat a pediatric condition (e.g., from twelve to six hour‐long outpatient appointments). From the CYP patient's perspective, this frees up a resource that could be used differently— here, time that can be devoted to other (paid or unpaid) activities. In the context of an economic evaluation that is interested in capturing use of patients' resources (be they time or money), this is a gain that needs to be reflected in calculations. Conceptually, in all main types of economic evaluation, use of resources is translated into opportunity costs and is included on the “cost” side of calculations. Could there be situations where the same change in time is inadvertently reflected as both a cost and an outcome? For example, when concerned with time in treatment could the gain of saving an hour of a hospital appointment also be reflected on the outcomes side of the evaluation, which might lead to double‐counting? For this to be a concern, a number of conditions would need to be met. First, outcomes of the evaluation would have to be measured through health outcome measures that can, at least in theory, adequately and appropriately capture the utility brought about by a shorter appointment. Equally restrictively, timing will need to be opportune; for instruments that aim to capture such outcomes at a particular point in time (e.g., the day the instrument is administered), the gain in time due to a shorter appointment will need to have taken place on that particular day. Last but not least, such concerns would be relevant only to economic evaluation that can accommodate such outcomes, such as cost‐utility analyses.

For these reasons, double‐counting is unlikely when concerned with CYP's time spent receiving treatment. However, it is conceivable that changes in time as a result of an intervention may be, to some extent, captured by health‐related quality of life measures. Whether this is the case, as well as under what circumstances and for which health care measures or dimensions this may be relevant, are pertinent questions for further research.

### 4.3 How Can We Identify a Value for CYP's Time?

Attaching a monetary value to CYP's time is a significant challenge, with difficulties arising from the fact that CYP's time is not traded in the labor market. However, there has been no shortage of suggestions about possible estimates of the monetary values of interest. A recent article reviewed the literature to identify such estimates and discussed their perceived strengths and drawbacks (Andronis et al. 2019). Key approaches used to arrive at such estimates—the ones we deem to be most suitable or least problematic—are discussed below.

One possible way is to infer the value that CYP place on their time through revealed preference methods. Such methods have been used in a variety of contexts and areas, including in transportation, environmental, educational and health economics, in some cases to elicit the monetary value of individuals' time (Dalenberg et al. 2004; Feather and Shaw 1999; Fezzi et al. 2014; Larson et al. 2004). However, situations where one can observe the shadow price of time are rare (Hunt 2019) and we are not aware of empirical findings related to CYP's time in the literature. In addition, findings can be crude and limited by the exact conditions of the transaction. Indeed, unless one can modify the terms of a transaction, there is limited opportunity to identify how preferences vary across different situations and categories of respondents (Feather and Shaw 1999).

Stated preferences elicitation methods can offer a solution to this. Such methods seek to deduce the value individuals place on different goods or services by presenting them with carefully constructed hypothetical scenarios and asking them to state their preferred course of action. On this premise, it is possible to tease out the value individuals place on their time by asking them to consider different scenarios, and this can be done through various methods, most prominently contingent valuation and discrete choice experiments (Louviere et al. 2000). Such methods have been used extensively in different fields (e.g., in transport economics, to estimate the monetary value of travel time) (Bates 1988; Börjesson and Eliasson 2014; Hensher 2001; Hess et al. 2017; Truong and Hensher 1985) and are increasingly used in health economics, including in some cases, for the purpose of estimating the value of time (Borisova and Goodman 2003; Hoefman et al. 2019; Neumann et al. 2016; van den Berg et al. 2017; Verbooy et al. 2018).

At the heart of contingent valuation methods are two Hicksian consumer surplus measures: willingness to pay (WTP) for purchasing or using a good, and willingness to accept (WTA) as compensation for foregoing a good (Mitchell and Carson 2013). The former is appropriate in cases where individuals consider purchasing or getting access to the use of a good, while WTA is suitable in situations where an individual is being asked to forego the use of a good (Carson 2000). Other methods include conjoint analyses (typically, exercises that ask respondents to rank or rate alternatives with respect to their degree of preference) and discrete choice experiments (commonly, experiments that ask respondents to choose between alternative options characterized by specific attributes) (Lancsar et al. 2007; Louviere et al. 2010). The latter have attracted increasing interest and have found application in different fields of enquiry, prominently transportation and health economics (Clark et al. 2014).

Stated preference techniques have been proposed to infer the “unit cost” of patients' time use in economic evaluations (Gold et al. 1996; Neumann et al. 2016; Sendi and Brouwer 2004). As a way of identifying the value of time for adults unable to work and for children, (Neumann et al. 2016) suggest that one could elicit willingness‐to‐pay for time inputs, in the same way that weights can be derived for health states. Perhaps unsurprisingly, there are also a number of applications with a focus on eliciting and inferring the value of one's time for use in economic evaluation (Lascelles 2008; Portrait et al. 2019 ; Randriamaro and Cook 2022). Such approaches offer more granular information: by asking respondents to state their preferences over different combinations of attributes related to time, including a monetary attribute, one can infer how the marginal value of time varies in different situations. Downsides of these methods include the effect of income (or the absence of it) on WTP/WTA values, and the complexity of ranking or choosing alternatives defined by numerous (and in some cases, intangible or unfamiliar) characteristics. This is particularly true when the focus is on CYP respondents (especially pre‐adolescents) who may not have the cognitive capacity or experiences to provide valid answers, or give stated preference answers that agree with and reflect “true” (revealed) preferences, especially when these relate to money (Valkenburg and Cantor 2001).

Other approaches are, at least in theory, also possible. One way would be to infer the actual value of children's time by linking educational absence to attainment and, subsequently, loss of future earnings (Gottfried 2010; Stanca 2006). However, unless the interest is in identifying the monetary value of greater lengths of time (e.g., due to avoiding prolonged ill health), this approach would offer a blunt instrument and estimates that are unlikely to capture the effect of short‐time losses. Alternative approaches, based on subjective wellbeing (or life satisfaction), have also been proposed for non‐market valuation (Fujiwara and Campbell 2011). These approaches use econometric methods to estimate the life satisfaction provided by non‐market goods, and this is then converted into a monetary figure by also estimating the effect of income on life satisfaction, but these are subject to similar restrictions as wage‐based approaches.

It is important to note that all these approaches are limited by uncertainties, not least whether non‐adults (especially pre‐adolescents) have the necessary understanding or experiences to provide valid answers, particularly when these relate to money (OECD 2006; Valkenburg and Cantor 2001). We look at this in more depth in the next section.

### 4.4 Whose Preferences Should Count?

Any attempt to derive the value of a good or service will have to tackle the important question of whose preferences should be sought and used as the basis for valuation. This question has attracted much attention in research aiming to value health outcomes in CYP and has led to useful insights, some of which may also be applicable to the valuation of CYP's time (Brazier et al. 2005; Lipman et al. 2021; Ramos‐Goñi et al. 2022; Ungar 2011).

In its most basic form, this is a question about whether the preferences taken into account should be those of affected individuals (in this case CYP) or of other members of society. From a theoretical point of view, it has been argued that affected individuals are the best judges of their preferences and, thus, they should be the source of valuation (Pigou 1951). In applied cost benefit analyses, it has been epitomized in discussions about who has “standing”, that is, whose welfare is to be affected by the evaluated course of action (Boardman 2008). On this basis, CYP should be the entities whose preferences should be sought and accounted for. For example, Mishan and Quah (Mishan and Quah 2020) lend support to individuals' valuation of their time (here, leisure time): *“What we call his leisure may be complete idleness or it may be, wholly or in part, recreational, educational or productive. But whatever he chooses to do with this ‘leisure’, the economist has to accept the person's own valuation of it in calculating his opportunity cost.”*

Adopting the perspective of the affected individual is not uncontested, especially as it is very likely that the value of time—for example an hour spent in a particular activity—may be weighted differently by different entities, for instance CYP, their parents or guardians, and the wider public. Activities that are generally perceived as highly beneficial from society's viewpoint may be seen as less valuable (or enjoyable) by CYP. Views may also diverge between members of the public who do, and do not have, parental responsibilities. This question has been explored in relation to valuation of health outcomes (Åström et al. 2022; Marshall et al. 2016) and environment‐related health risks (Alberini et al. 2010; Dardanoni and Guerriero 2019), though, to the best of our knowledge, it has not been explored in the context of CYP time valuation for economic evaluation. Time spent in formal education offers an apt example. Education is beneficial to society: schooling is a key means of building human capital, which brings about economic growth and prosperity (Schultz 1961; Solow 1956) and results in non‐pecuniary benefits (e.g., reductions in criminal behavior, greater political participation and greater sense of citizenship) (Lindsay 1984; Machin and Vignoles 2005; Vila 2000). In contrast, most CYP attach a lower value to activities categorized as committed time, such as school time (Gershuny and Sullivan 2019; Griffiths 2011). Thus, depending on whom to ask, it may well be that an hour of formal education forgone is assigned a greater or lesser weight, and preferences of one party are perceived as welfare‐decreasing for the other. We return to this point in the following section.

A further argument questions whether CYP have preferences that are “valid”. This is often judged by determining the degree to which preferences adhere to axioms of consumer theory. As a minimum, this requires that decision‐makers are rational and have preferences over a set of possible choices that are complete and transitive (Harbaugh et al. 2001; Lancsar and Louviere 2006). Experimental research suggests that CYP do exhibit rationality and strategic thinking (Bereby‐Meyer et al. 2004; Harbaugh et al. 2001; Sutter et al. 2019), however the degree to which this is the case is likely to vary across different age groups and contexts. Linked to this is the question of whether CYP have the experience to judge unfamiliar situations. While this is an important concern when CYP are asked to judge conditions (e.g., health states) that they may have never experienced, this is, arguably, less of an issue in expressing their preferences about time spent in different activities and situations. Similarly, ethical issues related to asking children to face questions related to death—which is a substantial concern in outcomes valuation—are less applicable to time valuation. In addition to making decisions on how to spend their time, recent studies have found that children as young as 7–8 years old are familiar with money (Berti and Bombi 1988), start to understand that it can be exchanged for goods (Gasiorowska et al. 2012) and, in some cases, they can trade‐off money for benefits (Dardanoni and Guerriero 2019; Guerriero and Cairns 2017). However, we advocate that gaining insights into how CYP think about money (e.g., constraints and levels in relation to allowances or parents' income) should be a key first stage in research aiming to elicit monetary values for CYP's time.

### 4.5 What Perspective Should (Proxy) Respondents Take?

Assuming that CYP have “valid” preferences, a subsequent question is whether they are able to express them. Reasonable questions are often asked about the degree to which CYP may be able to engage with common preference elicitation techniques, which are often seen as cognitively demanding. While recent findings of empirical research suggest that CYP can successfully engage with stated preference elicitation exercises (Barber et al. 2019; Dalziel et al. 2020; Dardanoni and Guerriero 2019; Mott et al. 2019; Ratcliffe et al. 2011), this typically relates to older CYP and is drawn from a different context—namely, preferences for health outcomes. There are at least two reasons why younger children may be unable to engage with preference elicitation tasks: the complexity of such tasks (Bereby‐Meyer et al. 2004) and limitations in their capacity to engage with questions around concepts that are not fully understood. For example, it is known that young children have difficulties in judging concepts of relevance to this exploration, such as lengths of time (Droit‐Volet et al. 2001; Gautier and Droit‐Volet 2002).

If younger children's preferences cannot be expressed, they will need to be sought from other informants (e.g., older children, adults). This gives rise to questions about who should be asked to provide answers, and whose perspective (or point of view) they should be asked to take. Different possibilities in relation to respondent and perspective are illustrated in Table 1. Rows denote possible groups of respondents and columns show the perspective they could be instructed to take during a valuation exercise. These result in five distinct cells. Cell A1 reflects arrangements where adults only are asked to value CYP's time from their own perspective. On the face of it, this may be seen as a straightforward and uncontroversial situation, especially on the basis of arguments that this group contributes to—and therefore should exert some control over—the resources to be allocated. This, however, would disenfranchise CYP who are capable of expressing their views, something that would be rightly seen as undesirable (British Academy 2022; United Nations 1989). Cell A2 represents a situation where only adults are asked to respond, but they are explicitly asked to take the perspective of CYP (i.e., answer as if they were CYP). In such a situation, adults need to be specifically told what age of CYP they should consider. A possible solution to this is for different randomly selected adults in the sample to be asked to assume the perspective of CYP of a particular age or age group (e.g., < 7 years old, 7–11 years old, 11–18 years old). However, adults' preferences will inevitably be influenced by their views and experiences, which offers no guarantee that their judgments will converge with those of CYP. Recent literature points to differences in preferences expressed when adults are asked to consider their or a CYP's perspective, albeit this relates to preferences for health outcomes (Lipman, Reckers‐Droog, Karimi, et al. 2021; Reckers‐Droog et al. 2022).

**TABLE 1:** Matrix showing possible combinations of respondents of valuation exercises and perspectives these respondents could be asked to adopt.

	Perspective^b^	
Respondents^a^	Own (adults = adults; CYP = CYP) (1)	CYP (adults = CYP, CYP = CYP) (2)
Adults only (A)	A1	A2
Capable CYP only (B)	B	
Adults and capable CYP (C)	C1	C2

^a^ Rows denote possible groups of respondents.

^b^ Columns show the perspective different groups of respondents could be instructed to take during a valuation exercise.

Cell B is based on the premise that, as long as they are capable of forming and expressing their views, the best persons to ask are CYP themselves. This is an appealing proposition, as CYP would be drawing on their own experiences on judgments that affect them (they have “standing”) and is relatively uncontested insofar as CYP are asked to express preferences related to an age band within which their age falls. Complications arise when individuals in this group are asked to take the perspective of CYP of other ages, especially younger children. In such a situation, the inevitable issue of asking people to make judgments (“guesses”) on behalf of other people comes into play.

Cells C1 and C2 reflect a situation where a combined sample of adults and CYP are asked to respond. This has been put forward and discussed in the literature on valuing CYP's health outcomes and, although it is a credible idea, there are issues in operationalizing this, not least with constructing an appropriate sample (Rowen et al. 2022, 2020). C1 reflects a situation where a sample comprising capable‐to‐answer CYP and adults would be asked to express their preferences, each from their own perspective. This would avoid asking people to make choices on the basis of someone else's perspective, though only a fraction of the preferences elicited from this sample would be actual preferences of CYP. In contrast, C2 denotes the case where this combined sample would be asked to state their preferences from the viewpoint of CYP, which raises the same concerns as any arrangement that asks people to consider the points of views and preferences of others. A potential solution to these challenging questions may be to recognize that the views of different entities are valuable, and combine these using agreed weights.

## 5 Moving Forwards

Over the last few decades, the increasing interest in making sure that public resources are spent prudently has led to considerable efforts to advance the methodology for carrying out economic evaluation in health care. In parallel, there has been a notable drive to adapt and develop methods suitable for use in evaluations of interventions for CYP (Petrou 2022; Ungar 2009).

However, uncertainties and gaps remain in both our understanding and in our methodological “playbook”, including how best to account for the opportunity cost of CYP's time in economic evaluations conducted from a societal perspective. To approach and resolve these, it is useful to draw up an agenda for research.

First, research is needed on ways to improve the available tools for measuring differences in CYP's time spent in treatment across interventions. While some data can be obtained from routine sources (e.g., routinely collected records about hospital appointments), much of the information required will need to be collected directly from individuals. Unlike for adults, there has been a paucity of “resource use measures” containing a standardized and validated set of questions appropriate for obtaining such information, either directly from CYP or from their parents/guardians/carers. Developing a questionnaire that is aptly tailored to CYP and appropriately comprehensive would be highly useful, and progress has now been made towards this goal (Morgan et al. 2026). The next logical step would be to identify dependable ways of translating changes (gains or losses) in the resource “time” into monetary units. To do so, we need a better understanding of how CYP think about their time, its value and its defining attributes (e.g., committed or discretionary time), how this varies across age groups and whether respondents are comfortable with answering questions that ask them to trade time for money. For this, it is important that researchers engage with CYP from the first steps of the exploration to discuss and present different approaches, check their suitability and cognitive burden, and test possible adaptations to standard techniques (e.g., combinations of different elements of valuation approaches). Meanwhile, in the absence of empirical estimates of the value CYP place on their time, we feel it would be helpful for analysts to choose and use “interim” values—provided these choices are justified, open to scrutiny and tailored to context and the age of the CYP in question. For example, Neumann et al. (2016) suggest using wage rates for adolescents participating in the labor market as proxies for the value of older CYP's time. At a minimum, any differences in time demands placed on CYP across alternative care programmes should be clearly described by the analyst and communicated to decision‐makers, even when these cannot be monetized.

Inevitably, empirical research alone will not be able to address all uncertainties. Normative questions, such as at what point a child's time has a discernible opportunity cost and whose views should valuation reflect or combine (e.g., to what extent the views of parents or carers should be incorporated in valuations and whether preferences of decision‐makers ought to be taken into account) can be informed by empirical evidence, but they cannot be resolved—satisfactorily and in their entirety—by it. For this, there is a need for researchers (as well as decision‐makers and the public) to engage in open discussions and debate, and we feel that pinpointing challenges and highlighting important questions is a necessary first step.

## Funding

This work is part of a larger project funded by the National Institute for Health and Care Research (NIHR) through an Advanced Fellowship award (L. Andronis, NIHR301711). Views expressed are those of the author(s) and not necessarily those of the NIHR or the Department of Health and Social Care in the UK.

## Ethics Statement

The authors have nothing to report.

## Consent

The authors have nothing to report.

## Conflicts of Interest

The authors declare no conflicts of interest.

## Permission to Reproduce Material From Other Sources

The authors have nothing to report.

## Data Availability

Data sharing not applicable to this article as no datasets were generated or analzsed during the current study.
